# Growth Mechanism and Luminescent Properties of Amorphous SiO_x_ Structures via Phase Equilibrium in Binary System

**DOI:** 10.1038/srep30901

**Published:** 2016-08-01

**Authors:** Changhyun Jin, Seon Jae Hwang, Myeong Soo Cho, Sun-Woo Choi, Han Gil Na, Suyoung Park, Sungsik Park, Youngwook Noh, Hakyung Jeong, Dongjin Lee

**Affiliations:** 1 School of Mechanical Engineering, Konkuk University, Seoul, 143-701, Republic of Korea; 2 Inha Analytical Instrumentation Center, Inha University, Incheon, 402-751, Republic of Korea; 3 Sensor System Research Center, Korea Institute of Science and Technology, Seoul, 136-791, Republic of Korea; 4 Department of Materials Science and Engineering, Inha University, Incheon 402-751, Republic of Korea; 5 Advanced Materials and Manufacturing Technology, Inha University Business Incubator, Incheon 402-751, Republic of Korea; 6 Helmut-Fischer Korea, 462, Dogok-ro, Songpa-gu, Seoul, 05574, Republic of Korea

## Abstract

Balloon whisk-like and flower-like SiO_x_ tubes with well-dispersed Sn and joining countless SiO_x_ loops together induce intense luminescence characteristics in substrate materials. Our synthetic technique called “direct substrate growth” is based on pre-contamination of the surroundings without the intended catalyst and source powders. The kind of supporting material and pressure of the inlet gases determine a series of differently functionalized tube loops, i.e., the number, length, thickness, and cylindrical profile. SiO_x_ tube loops commonly twist and split to best suppress the total energy. Photoluminescence and confocal laser measurements based on quantum confinement effect of the embedded Sn nanoparticles in the SiO_x_ tube found substantially intense emissions throughout the visible range. These new concepts related to the synthetic approach, pre-pollution, transitional morphology, and permeable nanoparticles should facilitate progress in nanoscience with regard to tuning the dimensions of micro-/nanostructure preparations and the functionalization of customized applications.

The unique controlled morphology and wide optical emission properties of low-dimensional (in particular 0D and 1D) semiconductors make them one of the most sophisticated core technologies in optoelectronics^1^, photonics^2^,^3^, and plasmonics^4^,^5^. In particular, the significance of fullerenes^6^ has inspired much systematic curiosity in many artificial periodic patterns such as strings of beads^7^, chains^8^, and bamboo-like tubes^9^ in nanostructures. These morphological peculiarities were accomplished by the following specific synthetic manipulations: metal particle migration induced by annealing^7^, stress induced by new carbon layer formation^10^, the periodic instability of catalyst particles^11^,^12^, and metal atom evaporation^8^. Most of these structures have been confined to carbon-based materials. The special case of spiral twisted morphologies as a patterning series has been considered a hot topic due to the unique design and physical/chemical identification compared with those of the other forms. Thus, many promising approaches for the synthesis of twisted materials have been studied for various potential applications such as liquid crystals, sensors, and optical activity^13^,^14^,^15^,^16^,^17^,^18^. However, despite the striking progress in twisted structures, most synthetic routes are limited to chemical-based reactions that include polymers, closely akin to self-assembly^19^,^20^. The development of physical procedures for the desired twisted morphology remains a challenge. To date, optical research on the luminescent efficiency of Si based not on amorphous nanoclusters (a-Si NCs) but on crystalline nanoclusters (c-Si NCs)^21^ has been carried out on the following: the formation of porous silicon^22^; implantation of Si^+^ ions^23^; laser ablation^24^; sputtering of Si^25^, SiO^26^, and SiO_2_^25^; and structural evolution via thermal annealing^27^. In most cases, however, the main luminescent techniques of Si-based materials have been focused on the production of Si nanocrystals or transformation from amorphous to crystalline structures. Therefore, innovative new methods dissimilar to conventional methods are required to highly enhance the optical efficiency and range of Si-based materials.

In this paper, we present a solution to the above problems, namely, well-designed twisted micro-/nanotube morphology via a physical reaction basis and a luminescent origin through the embedment of metal instead of c-Si NCs. Our synthetic route, which we call direct substrate growth (DSG), is novel in that it is a one-step process that requires no direct source, catalyst, and post-treatment. In addition, it has the advantages of size-controllable one-dimensional (1D) tube formation and emission spectra throughout the visible range at the same time. Our original concept is that the main factors that influence the final morphological and optical properties depend mostly on the selections of the pre-contaminated concentration and embedded nanoparticle size.

## Results

**Figure 1:**
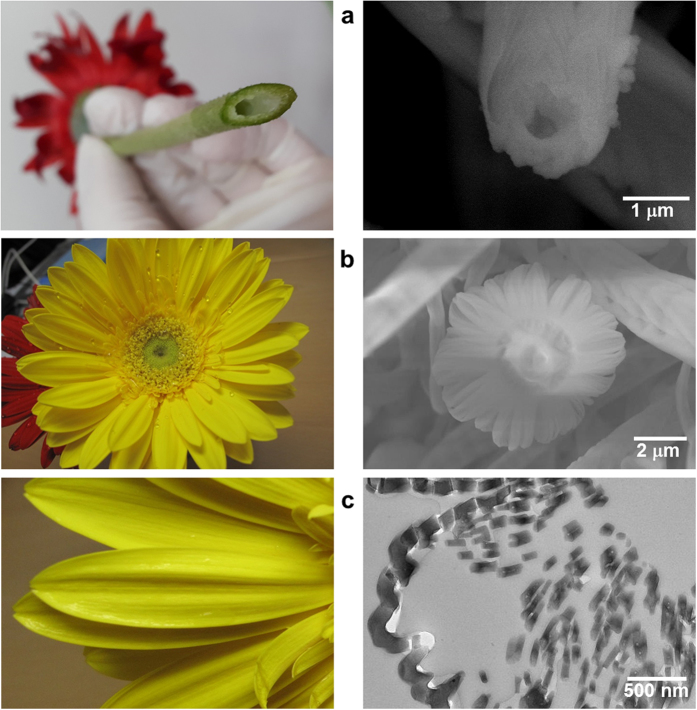
Real flower (gerbera) and corresponding SEM and TEM images: (**a**) SEM image showing a hollow SiO_x_ structure with overlapping scale-like patterns on the surface, (**b**) SEM image of a typical flower-like SiO_x,_ and (**c**) TEM image of flattened loops similar to the petals of flower-like SiO_x_.

In this study, tubular flower-like SiO_x_ embedded with Sn nanoparticles (~5 nm in size) was synthesized for the first time by using a Si substrate with assistance from In_2_O_3_ and graphite powders in pre-deposited SnO_2_ thin films. Figure 1 presents the main concept with an image of a real gerbera flower: (a) the basic concept of a hollow structure, (b) the flower-like morphology, and (c) the connected loops of the inner part of the petal. The detailed preparation procedure is described in Materials and Methods. An experimental study expressed the carboreduction related to SnO_2_ dissociation within the SnO_2_/C/Sn system as follows^28^:


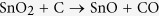



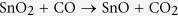



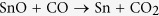



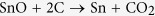


Based on the Si–Sn phase diagram^29^ (Supplementary Fig. S1), when the Sn nanoparticles are stuck in a Si substrate, the Sn circumference is encircled by neighboring Si atoms. At a glance, it seems that mass (Si) diffusion from the underlying substrate through the metal particles can reasonably explain the nucleation and growth mechanism^30^,^31^ because there is no other Si source except the substrate in this system. However, only diffusion of Si atoms is not sufficient to attain the supersaturation for Si nucleation at the contact points between Sn-edge and Si-surface in that a range of surface melting for Si substrate is too narrow (a few tens of degrees) to drastically lower the melting point of Si and to effectively induce Si diffusion. Accordingly, both nucleation and growth of SiO_x_ structures would be dominated by volatile SiO vapor generation from the Si substrate, especially at lower O_2_ pressures and higher temperatures (620–1000 °C)^32^,^33^ in agreement with our synthetic conditions (remnant O_2_ and 1100 °C). The vapor-phased SiO directly adsorbs onto the surface of Si-Sn or Si-O-Sn structures. The liquidized small-size ternary droplets, reacting with the surface of Si compared with bulk Si, facilitate more rapid nuclei formation, and hence continuous growth development^34^. In addition, SnO and Sn are mixed with In_2_O_3_, and the Sn^4+^ ions originating from the pre-contaminated SnO_2_ thick films are distributed on In^3+^ sites to facilitate the substitution of Sn^4+^ for In^3+ ^35. The additional effects of the roles of In_2_O_3_ powder and carrier gas are presented in Supplementary Fig. S2.

**Figure 2:**
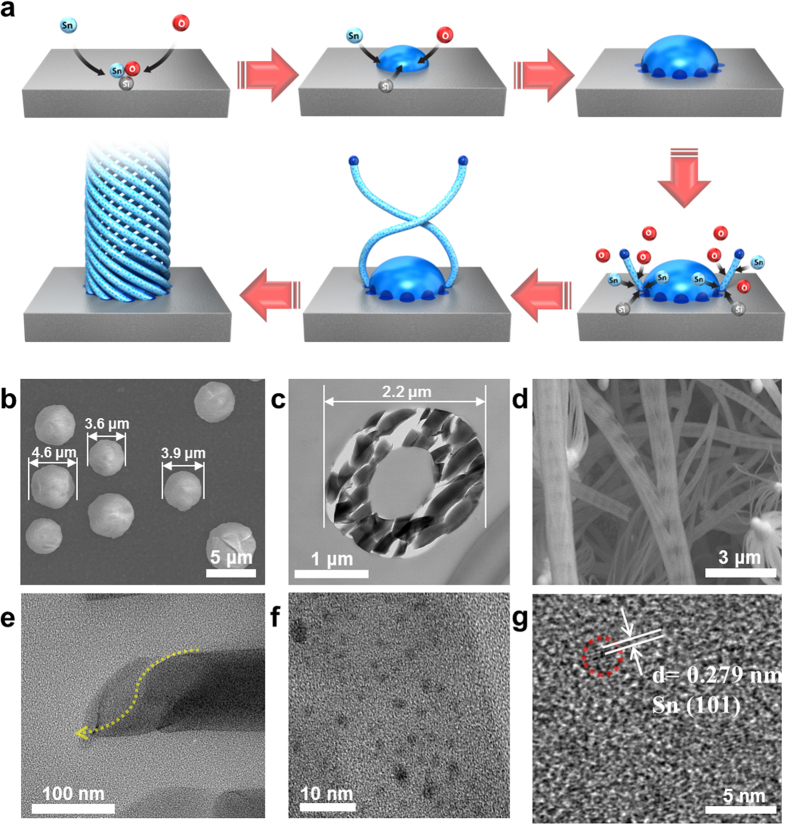
Schematic sequence of the formation of tubular 1D SiO_x_ and characteristic SEM and TEM images: (**a**) adsorption-nucleation-twisted growth procedure with a pre-contaminated SnO_2_ film in the tube furnace, (**b**) typical Sn particles (exact Si–Sn–O droplet on the Si surface), (**c**) cross-section of a SiO_x_ tube comprising SiO_x_ fragments, (**d**) typical SiO_x_ tube morphology, (**e**) a cut TEM image indicating the twisted SiO_x_, (**f**) enlarged Sn-embedded SiO_x_, and (**g**) an HRTEM image of crystalline Sn embedded in amorphous SiO_x_.

Figure 2 shows SEM images characterizing the process of the twisted growth mechanism for tubular flower-like SiO_x_. As shown by the schematic in Fig. 2a, Si atoms obtained from the Si substrate and the remaining O gas combine with Sn atoms originating from nanoparticles or the internal SnO_2_ film to form ternary (Si–O–Sn) droplets. However, based on the phase diagram (Supplementary Fig. S1), the ternary solid solution cannot include a Sn concentration higher than 0.1 at%, which suggests that the elemental composition of a droplet is mostly a mixture of Si and O. Supplementary Figure S3 confirms the existence of three main elemental compositions at the (a) tube and (b) tip regions. The low level of Sn incorporation into a ternary droplet results in a uniform dose distribution for the whole SiO_x_ tube. The initial interfacial reaction-controlled process at the smooth surface and the diffusion-controlled reaction at the rough surface occur consecutively. Once the SiO_x_ nanostructures nucleate at the ternary interface, the dominant individual SiO_x_ particles (e.g., ranging from 3.6 to 4.6 μm in Fig. 2b) start to grow. They keep the hollow SiO_x_ shape (i.e., no center SiO_x_) with the overall diameter mostly the same as (or smaller than) the Sn nanoparticle size (e.g., 2.2 μm in Fig. 2c). If a SiO_x_ tube is forced to realize a minimum diameter, it would likely follow a spiral configuration for an adjustable fit to make the smallest cylindrical volume (Fig. 2a). In other words, the natural tendency to suppress the total surface energy may maximize the bonding interaction, suggesting the formation of twisted surface morphology among numerous SiO_x_ loops grown from the circumference of the droplet (Fig. 2d). In particular, the torsion image in Fig. 2e may be used as clear evidence of our conjecture. Namely, the main reason for the helical surface pattern on tubular SiO_x_ seems to be connected with behavior to minimize the surface energy because our samples cannot be satisfactorily explained by other reasons such as crystallization^36^,^37^ and alignment^38^ owing to the instant effect. Figure 2f,g show the crystallized Sn-embedded amorphous SiO_x_. The influx of Sn nanoparticles is made possible through the diffusion of ternary droplets or dissociation of the SnO_2_ film. The Supplementary real-time Movie S1 supports the explanation of Sn embedment above. The Sn nanocrystallites are randomly distributed into the amorphous SiO_x_ (Fig. 2f), which implies no clear fringe, and produces the constant spacing in Fig. 2g. The interplanar spacing of Sn monocrystalline is 0.279 nm, which matches the (101) lattice plane of tetragonal Sn well (JCPDS 04-0673, lattice parameters: a = 0.5831 nm and c = 0.3182 (S.G I4_1_/amd (141)) (Fig. 2g).

**Figure 3:**
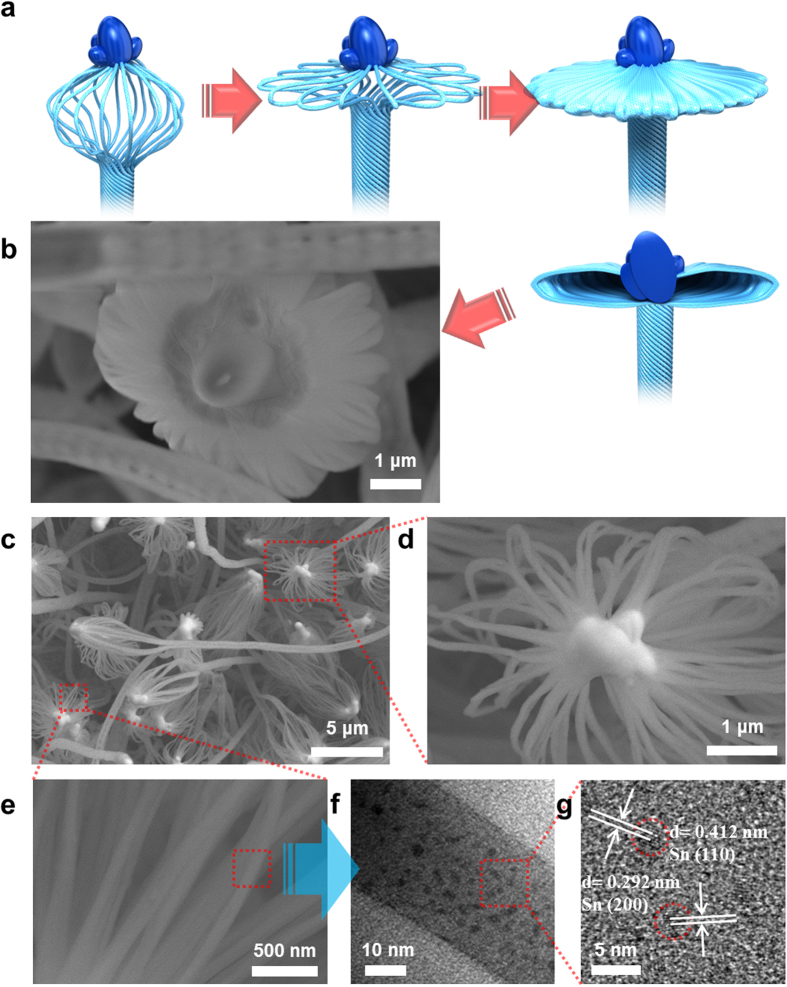
Schematic of the morphological transition from balloon whisk-like SiO_x_ to flat continuous aggregated loops and electron microscopic images of balloon whisk-like SiO_x_: (**a**) steps of morphological change arising from excess thermal and partial pressure, (**b**) typical flower-like SiO_x_ formation originating from the transition in (**a**,**c**) various balloon whisk-like SiO_x_ morphologies, (**d**) an enlarged image of the head part, (**e**) an enlarged image of loops in balloon whisk-like morphologies, (**f**) an enlarged image of the loop, and (**g**) an HRTEM image of (**f**).

When the respective SiO_x_ loops intersect with one another (Supplementary Fig. S4), the SiO_x_ generation series occurs in sequence to keep the front ellipse-shaped Sn head (Supplementary Fig. S4a). Occasionally, if two Sn heads are combined together, new SiO_x_ tubes grow, showing two SiO_x_ parts (Supplementary Fig. S4b). Then, the left- and right-handed directions are not particularly important. The SEM image in Supplementary Fig. S4b indicates that the large-sized Sn lump at the tip may act as a nucleation site for branched nanowires, just like Sn embedment into the SiO_x_ described above. In the case of balloon whisk-like SiO_x_ formation (Fig. 3a and Supplementary Fig. S4c), more than two SiO_x_ loops meet to combine at the tip. Sometimes, the Sn lump size is too large to lead to axial growth. Therefore, the extra thermal and pressured energy and source (SiO_x_) normally used for the SiO_x_ tube growth mechanism, are consumed by SiO_x_ nanowires diverging longitudinally or transversely between the Sn tip and SiO_x_ tube for some distance (Fig. 3b–d). This indirectly implies that many SiO_x_ loops are involved in SiO_x_ tube formation. Particularly, compared with the general twisted growth of SiO_x_ (Fig. 2e), these loops arising from balloon whisk-like SiO_x_ have no torsion and twist shape (Fig. 3e) owing to running out of surplus energy to split all loops. Figure 3f,g show enlarged TEM and HRTEM images, respectively, of the swollen unit SiO_x_ loop. Many different lattice planes such as (110) and (200) were found, which confirms that Sn nanoparticles are crystalline. After the flat flower-like formation of SiO_x_, the morphological transition accumulates regular patterns on the tube surface, as shown in Supplementary Fig. S5.

**Figure 4:**
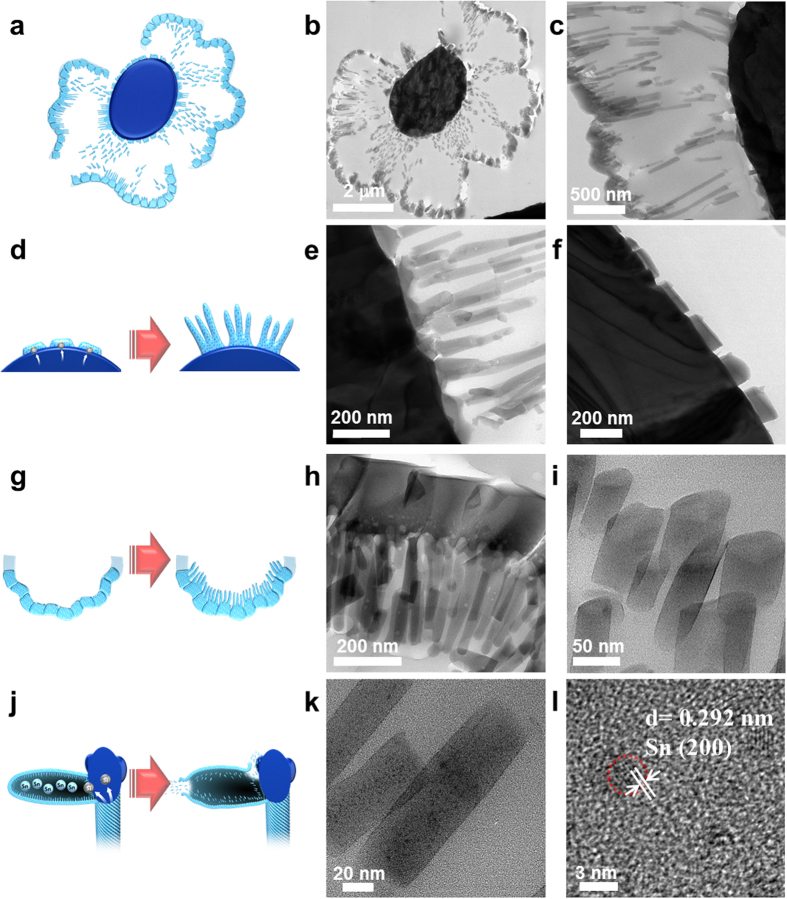
Schematic illustration and TEM images corresponding to individual regions in flower-like SiO_x_: (**a**) schematic illustration of SiO_x_ growths on both sides, (**b**) cross-sectional TEM image of flower-like SiO_x_, (**c**) enlarged loop part obtained from (**b**,**d**) nucleation and growth at Sn lump region, (**e**) boundary image of (**d**,**f**) cut SiO_x_ image originating from partial pressure differences, (**g**) nucleation and growth at the SiO_x_ wall region, (**h**) boundary image of (**g**,**i**) typical nano-SiO_x_, (**j**) Sn partial pressure in the closed loops, (**k**) Sn-embedded SiO_x_ nanowire remaining in the closed loops, and (**l**) an HRTEM of (**k**).

When the flower-like SiO_x_ is completely spread out, new and thinner SiO_x_ nanowires are created at both the center and edges (Fig. 4a–c): the center site is around the Sn lump (Fig. 4d–f), and the edge site is near the pre-formed outside SiO_x_ (Fig. 4g–i). In the first stage of nucleation and growth at the surface of the Sn lump, SiO_x_ can grow via combination effects mixing the solution–liquid–solid (SLS)^39^,^40^ and vapor–liquid–solid (VLS)^41^,^42^ mechanisms. The VLS mechanism somewhat differs from the SLS process in how the chemical source is introduced in the chamber. The former is carried out through the flow of gas, whereas the latter is performed via one-off means in a product^39^, even though the basic principles of using molten metal particles are similar. Based on our experimental procedure, a large number of Sn metals not only permeate into the ternary droplets centered on the Sn lump as a type of SLS growth but also adsorb onto the surface of the ternary droplets as a kind of VLS growth. On the other hand, the outside SiO_x_ wall also acts as a nucleation site at the same time as the Sn lump. Contrary to the evidence of nucleation and growth on the Sn lump surface, only the SLS growth system predominates instead of the VLS process because there are no remarkable tips in the newly produced shorter and thinner SiO_x_ nanowires. As described above, when all of the Sn-centered tips of the twisted SiO_x_ nanotubes encounter each other at one point, the twisted behavior is stopped and fixed. Therefore, extra energy is used to separate or expand the individual SiO_x_ loops. Both the number and split degree of loops increasingly become more severe to make an almost flat plane, even though there is some space in reality (left schematic illustration in Fig. 4j). The individual loops may combine into the whole SiO_x_ material without any disconnection to produce a closed system. Additional SiO_x_ nanowires may grow from both sides of the Sn lump and pre-formed SiO_x_ wall in this closed system. At this point of time, another possible point should be considered. If any defects including 0D, 1D, and 2D flaws occur on the preformed SiO_x_ walls (right schematic illustration in Fig. 4j), a large internal pressure produced by evaporation of the Sn lump would move from the larger partial pressure region (around the unflawed Sn part) towards the smaller partial pressure region (around flawed SiO_x_ part) to cause crystalline Sn (200)-embedded nanowires (Fig. 4k,l). The cross-sectional image in Fig. 4f indicates that sharp cutting planes come not from the pattern grown from the Sn lump but from a strong internal force. Here, the different lengths of SiO_x_ depend on how much new SiO_x_ nanowires are influenced by the internal vapor pressure of the Sn lump in the closed system according to the boundary condition, volume size, process duration, and kind of defects. The specific microstructural and compositional data for the behavior and arrangement/elemental composition of newly produced SiO_x_ nanowires in flower-like SiO_x_ sections are given in Supplementary Figs S6–8.

**Figure 5:**
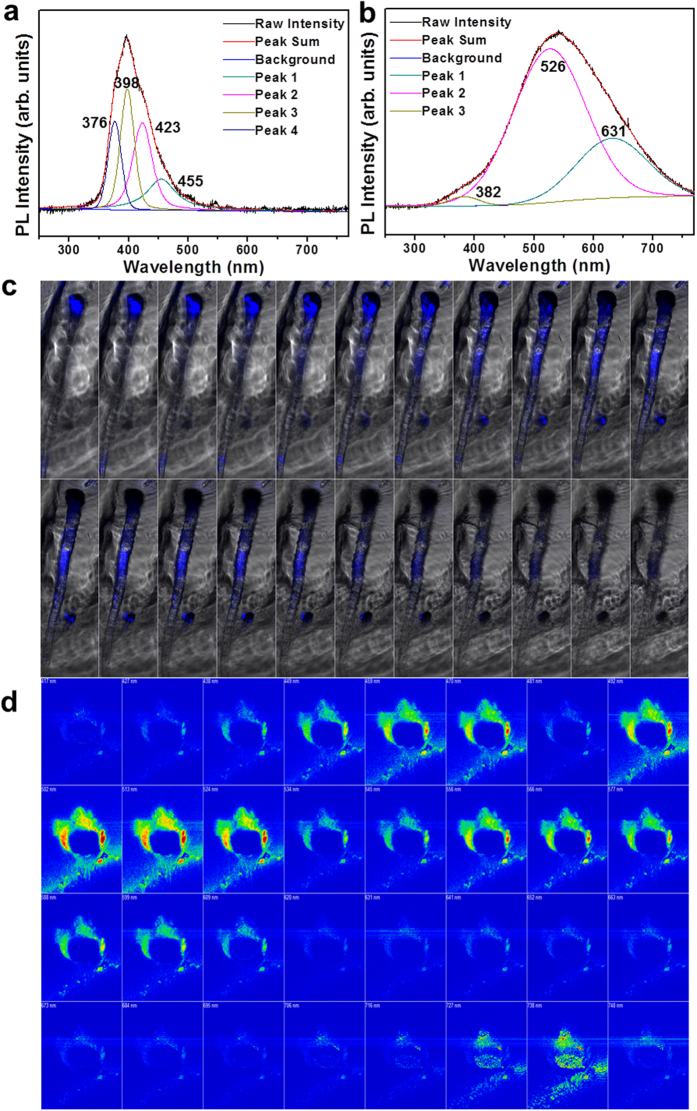
Optical properties of SiO_x_ nanotubes. PL spectra of as-synthesized Sn-embedded SiO_x_ tubes with Gaussian fit (**a**) at 397 nm and (**b**) at 540 nm. (**c**) Z-stack alongside the axial direction at the SiO_x_ tube region. (**d**) Confocal spectral image from 417 nm to 748 nm at the SiO_x_ flower region.

Figure 5 displays the normalized PL spectra of as-synthesized tubular SiO_x_ with embedded Sn (~5 nm) at room temperature. For the enlarged PL spectra, Fig. 5a shows a relatively narrow emission band centered at ~397 nm in the violet region corresponding to a Gaussian fitting consisting of violet emissions (376 and 398 nm) and blue emissions (423 and 455 nm). Figure 5b of the same samples reveals a relatively broad emission band centered around 540 nm in the yellow region corresponding to minor violet (382 nm), green (526 nm), and orange (631 nm) emissions of the Gaussian fitting. The other PL result is in Supplementary Fig. S9. No considerable PL emission peak in the SiO_x_ structure can be observed in the visible region (350–700 nm) apart from special functionalized Si quantum effects such as the strain^43^, Si nanoporous pillar array (Si-NPA)^44^, and molecular beam epitaxy (MBE)^45^. Thus, the specific violet and yellow emissions from tubular SiO_x_ in this work cannot be fully accounted for by SiO_x_ micro- and nano-tubes themselves because our samples consist of an amorphous structure, as shown in Figs 2 and 3. They may be caused by the embedment of Sn nanoparticles (~5 nm), which is known as quantum confinement effect (i.e., quantum dot)^46^,^47^. The energy band gap may be opened and tailored even in zero-gap materials analogous to graphene by confinement effects^48^,^49^. However, it is difficult to obtain a precise variability in the PL emission spectra over the samples because very precise control of the size and shape of Sn quantum dots in SiO_x_ is almost impossible. It is clear that the tolerance of various sized and shaped Sn quantum dots (i.e., less than 10 nm) to preserve the visible-range PL emission spectra is somewhat broad, as shown in Figs 2f,3f,4k and 5c,d. In other words, the wavelength and intensity of PL emissions obtained from different parts of samples depend very sensitively on the sizes and shapes of Sn quantum dots even though all our samples have broadly similar tendencies in the range of 380–400 nm and 530–540 nm. Consequently, the PL properties tend to depend on the PL sample collecting location in Sn-embedded SiO_x_ structures synthesized under the same process condition. Apart from the quantum confinement effect, the controlled hybridization of crystalline and amorphous structures would be another factor to enhance the PL properties. Li *et al*.^50^ reported that the PL emission of crystalline Sn and amorphous SnO_2_ nanoparticles reveals ultraviolet and blue emission due to the synergistic effect of the quantum size and interfacial electronic-coupling between the crystalline core (Sn) and amorphous shell (SnO_2_). The fact that the major emission range of Sn-embedded SiO_x_ tubes stretches from the longer wavelength (red emission) region to the shorter wavelength (violet emission) region, irrespective of the location of the sample (e.g., Z-stack at the tube (Fig. 5c) and confocal spectral imaging at the flower (Fig. 5d)), shows that there is a strong dependence on the size and shape of the Sn nanoparticles. The information on the subsidiary movie (Supplementary Movie S2, 3) offers further support for the continuous emissive transition of products at the tube and flower. Despite the remarkable strides in new synthetic techniques presented in this work, a complete understanding is still lacking. The effect on many process variables remains to be verified, such as the quantity of pre-contaminated SnO_2_ and supporting materials, synthesis temperature, process pressure, and duration.

## Discussion

The somewhat modified VLS technique surrounded with SnO_2_ films is fairly uncomplicated but very powerful for fabricating metal embedded tubular SiO_x_ by using the DSG without multiple artificial manipulations. The results indicate not only unique morphologies (twisted-, balloon whisk-like, and flower-like SiO_x_) but also optical properties throughout the visible range based on the combination of Sn heads and control of Sn particle sizes and shapes. The morphological transition is based on the pre-contaminated concentration, while the wide-ranging emissions originate from quantum confinement effect. This synthetic procedure is of tremendous interest for all kinds of innovative and progressive applications in nanoscience and nanotechnology.

## Methods

The fundamental synthetic steps for SiO_x_ tubes follow the typical vapor–liquid–solid (VLS) mechanism^51^,^52^. The only exceptional difference between our technique and the traditional method is whether or not the pre-contaminated metal oxide thick film is present before the production of target SiO_x_ tubes from p-type Si (100) substrates. Preformed SnO_2_ thick films may be able to provide the embedment of minute Sn nanoparticles into SiO_x_ tubes due to the carbothermal reductive dissociation of SnO_2_ for SnO and Sn over 1050 °C^28^. In our previous paper, we presented the growth factor and process variables for SnO_2_ film, as no specific substrate, onto the surface of alumina tubes^53^. The same procedure was repeated up to 10 times to supply the satisfactory amount Sn nanoparticles. After SnO_2_ formation to an adequate thickness, thermal evaporation was carried out with a mixture of In_2_O_3_ (1 g) and graphite (0.1 g) powders in the alumina boat by putting a Si (100) substrate upside down on the ceramic boat. Both In_2_O_3_ and graphite acted as supporting materials for the substitution for Sn and creation of CO/CO_2_ gases, respectively. The detailed process variables are as follows: The process temperature was fixed to 1100 °C for 40 min. An inlet gas of Ar (200 standard cubic centimeters per minute (sccm)) without any additional reacting gas was introduced into the chamber to make the total pressure in the tube 0.1 Torr (1 Torr = 1.333 × 10^2^ Pa). To compare the effects of the supporting materials (In_2_O_3_ and graphite) and used gas (Ar), the same cycles were reproduced with two different types: no In_2_O_3_ and graphite powders (a) with Ar gas (b) without Ar gas. Finally, when the furnace temperature was cooled to room temperature (RT), white-like SiO_x_ products were collected.

A field emission scanning electron microscope (FESEM, Hitachi S-4200, 20 kV) was used to characterize the surface morphology of SiO_x_ tubes, and a field emission transmission electron microscope (FETEM, JEOL JEM-2100F, 200 kV) was used to observe the local microstructures inside and outside the samples. High-resolution TEM (HRTEM) measurements were performed at confined areas in the thin products (70 nm) pre-cut via the ultramicrotomb (MTX/RMC) after the scratched samples were embedded and trimmed in the capsule with epofix (EMS). The emissive origin of Sn-embedded tubular SiO_x_ was analyzed according to the photoluminescence (PL, Maple) by using a He–Cd laser (325 nm, 55 MW) and confocal laser scanning microscopy (CLSM, LSM 510 META) with a diode-laser (405 nm, 30 mW) at RT. For the individual longitudinal and spectral properties, both Z-stacks generated by incrementally stepping and spectral imaging from 417 nm to 748 nm by META detector techniques were executed.

## Additional Information

**How to cite this article**: Jin, C. *et al*. Growth Mechanism and Luminescent Properties of Amorphous SiO_x_ Structures via Phase Equilibrium in Binary System. *Sci. Rep*. **6**, 30901; doi: 10.1038/srep30901 (2016).

## Supplementary Material

Supplementary Information

Supplementary Movie S1

Supplementary Movie S2

Supplementary Movie S3
