# Supplementary material for: Growth Mechanism and Luminescent Properties of Amorphous SiOx Structures via Phase Equilibrium in Binary System
Source: Sci Rep. 2016 Aug 1;6:30901. doi: 10.1038/srep30901 (PMC4967906; doi:10.1038/srep30901)
Supplement: Supplementary Information [file srep30901-s1.doc]

Supplementary Information

**Growth Mechanism and Luminescent Properties of Amorphous SiO_x_ Structures via Phase Equilibrium in Binary System**

*By Changhyun Jin, Seon Jae Hwang, Myeong Soo Cho, Sun-Woo Choi, Han Gil Na, Suyoung Park, Sungsik Park, Youngwook Noh, Hakyung Jeong, and Dongjin Lee**

**Effect of supporting materials and carrier gas**

Using the In_2_O_3_ source as supporting materials is necessary to synthesize SiO_x_ tubes, even if the assisted materials are not included in the final products due to the rapid substitution of Sn^4+^ for In^3+ 35^. Under certain specific conditions without In_2_O_3_ powder (Fig. S2), we found that the Si substrate was damaged (Fig. S2a–c, e) and general nanostructures were created (Fig. S2d, f). Overall, very local circular shapes that were similar to micro-scale craters were formed. This implies that many Sn aggregations rather than individual Sn nanoparticles influence the surface by facilitating the nucleation and growth of SiO_x_ nanostructures. The probability of SiO_x_ formation was also low. Enlarged SEM images (Fig. S2d, f) indicate that the worm-like as-synthesized SiO_x_ nanostructures were quite short and had lengths of a few tens of diameters or a few hundred microns. Therefore, the In_2_O_3_ source acts not only to promote the possibility of nucleation but also the reaction rate of the growth over the Si substrate. The other fundamental question for carrier gas (Ar) is how it behaves for stable SiO_x_ to be achieved. Figure S2 displays the morphology of the two different SiO_x_ nanostructures synthesized with (Fig. S2b, d, f) and without (Fig. S2a, c, e) carrier gas. The damaged morphologies of the Si substrate grown without Ar gas was characterized by retaining the evidence of a partly unbalanced incomplete Si surface, while the developed morphologies of SiO_x_ produced with the Ar gas were distinguished by active growth at the favorable nucleation sites in the crater. The extremely different flow rates of the carrier gas (Ar) on the morphologies of SiO_x_ nanostructures can be categorized into nanocluster- and nanowire-growth modes. With no carrier gas (Fig. S2a, c, e), rare disordered nanoclusters appeared on the substrate. When the Ar gas flow rate was present, nanowire growth occurred (Fig. S2b, d, f). That means that the carrier gas flow rate plays a key role in the transportation of the grown constituents and their effect on the structural morphology. However, the most critical issue among the many process variables is the indirect supply of the Sn metal from pre-deposited metal oxide films, not the direct provision of the source material like in the normal evaporation method.

**Behavior and arrangement of newly produced SiO_x_ nanowires in flower-like SiO_x_**

The TEM analysis (Fig. S6) showed that the countless SiO_x_ nanowires formed from the Sn lump and SiO_x_ walls are arranged in a SiO_x_ flower. The low-magnification TEM revealed large differences (Fig. S6a, b): the outside has thicker and longer SiO_x_ (a few hundreds of nanometers), and the inside has thinner and shorter SiO_x_ (a few tens of nanometers). The enlarged TEM images in Figure S6c–h exhibit the microstructural properties of the inner SiO_x_ flower. Based on Figure S6c, d, for the growth from a Sn lump, SiO_x_ nanowires (~20 nm) coming from the Sn surface move toward the outer loop tip after vacating the Sn lump (Fig. S6c, d, g) due to the higher partial pressure in the center. The light images of the Sn surface (marked by the red circles) satisfy this assumption and show traces of evidence. On the other hand, Figure S6e, f, h show the SiO_x_ nanowires grown from the SiO_x_ walls. Therefore, it is possible to nucleate and grow SiO_x_ nanowires on both sides at the same time due to the existence of Sn particles. Figure S7 indicates the various arrangements in the space between the center (Sn lump) and edge (SiO_x_ walls) with occasional nanoholes as shown in Figure S7c, d (marked by the yellow circles) and nanotubes as shown in Figure S7e (marked by the red circles).

**Elemental composition of newly produced SiO_x_ nanowires in flower-like SiO_x_**

The dots of the Sn nanoparticles in the enlarged TEM (Fig. S8a, b) and HRTEM (Fig. S8c) images in the flower-like SiO_x_ indicate that Sn was monocrystalline, while the absence of fringes in the SiO_x_ nanostructure indicates that SiO_x_ was amorphous. The interplanar spacing of the adjacent fringes was 0.29 nm, which agrees with the (200) lattice plane of tetragonal Sn having lattice parameters of a = 0.5831 nm and c = 0.3182 (S.G I4_1_/amd (141), JCPDS 04-0673). The additional HRTEM image also clearly reveals that the embedded Sn nanoparticles were randomly distributed over a large area in the SiO_x_ structure. The energy dispersive X-ray spectroscopy (EDX) results (Fig. S8d) showed that, despite the amorphous structure throughout, the Sn nanoparticle region covered with the SiO_x_ nanostructure consisted of Si, Sn, and residual O (as well as C and Cu resulting from the TEM sample preparation). Thus, the elemental compositions of the pre-produced SiO_x_ (micro-scale SiO_x_) and newly produced SiO_x_ (nano-scaled SiO_x_) were almost the same.


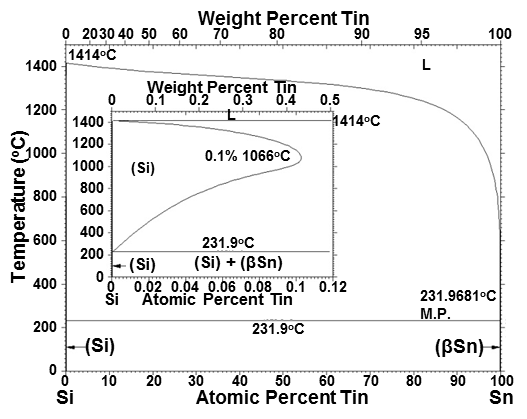


**Figure S1.** **Assessed Si–Sn phase diagram based on atomic and weight percent of tin.**


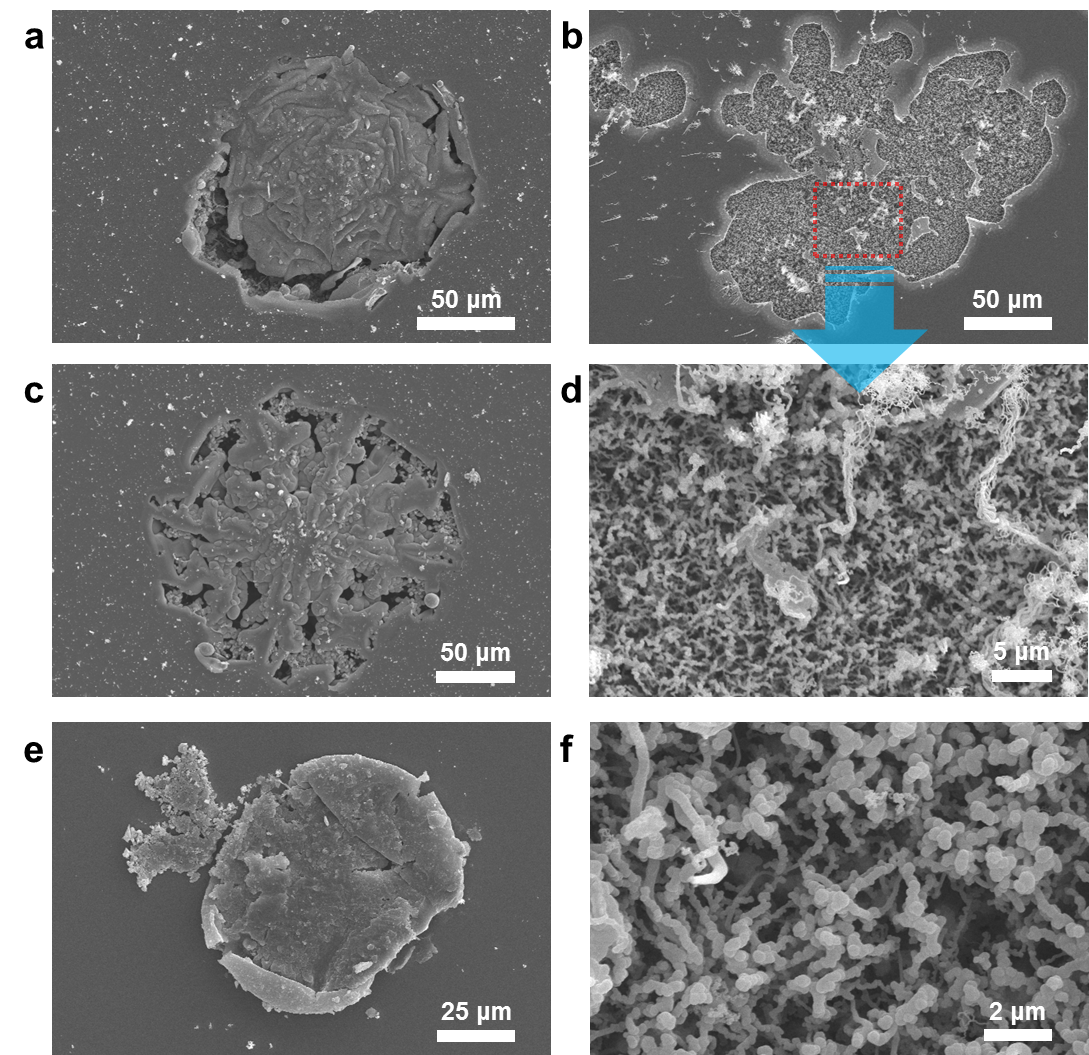


**Figure S2.** **SEM images of influence by carrier gas (Ar) with no supporting In_2_O_3_ powder**: (**a,c,e**) no carrier gas conditions, (**b,d,f**) 200 sccm carrier gas conditions. (d) and (f) are magnifications of (b) and (d), respectively.


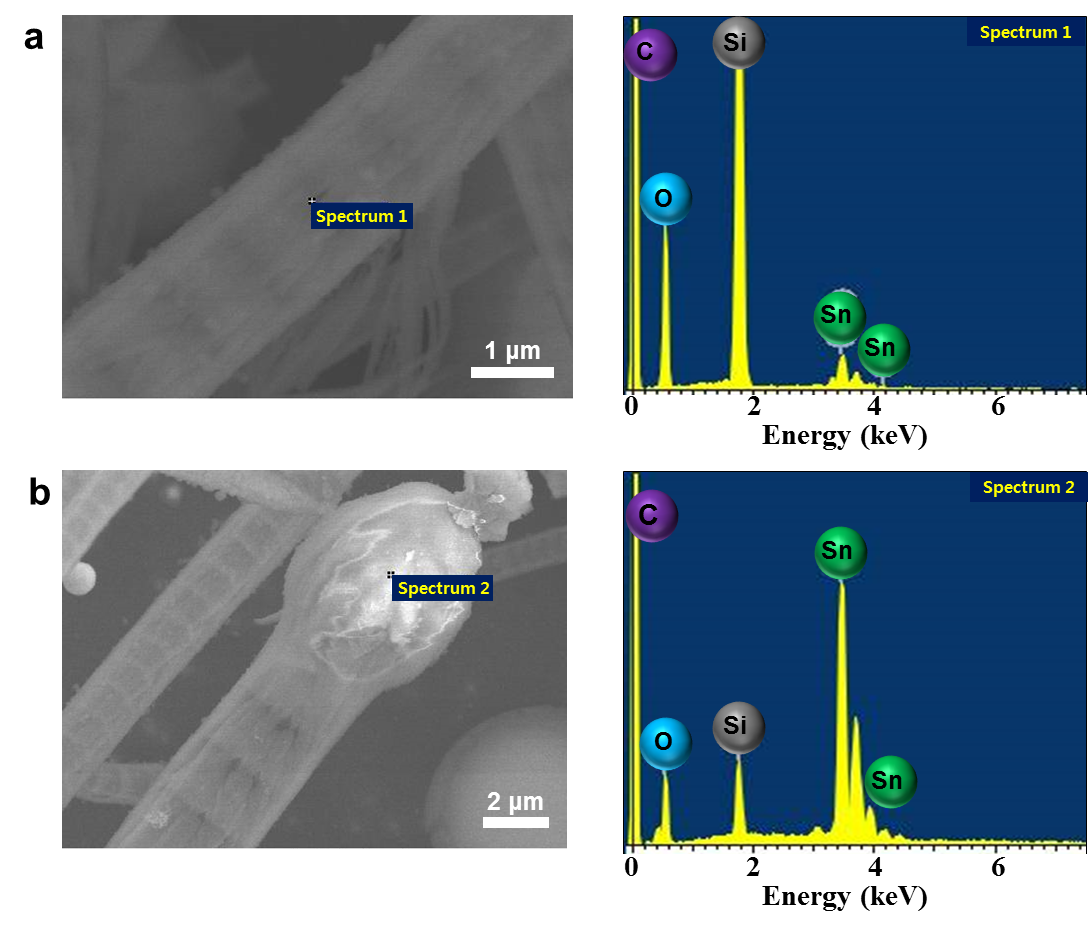


**Figure S3.** Elemental compositions at the (**a**) tube and (**b**) tip of the tubular SiO_x_ structure.


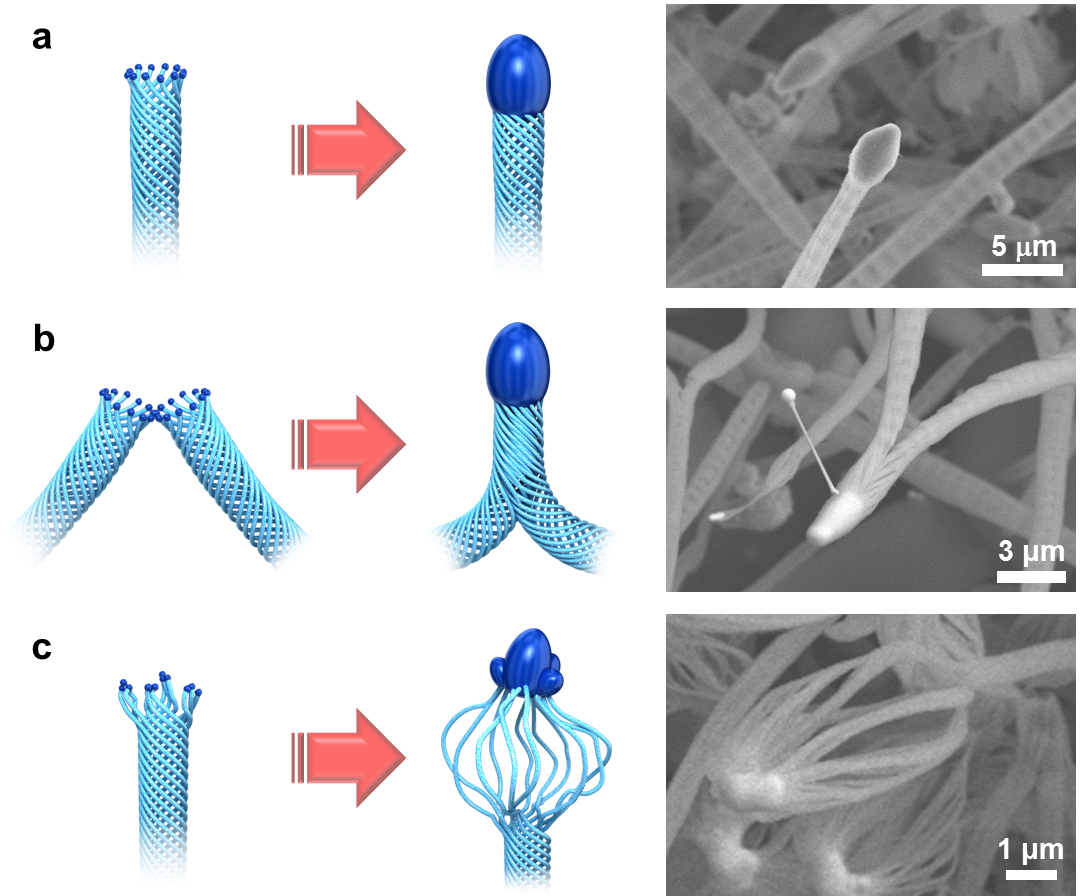


**Figure S4.** **Various SiO_x_ schematics and SEM morphologies**: (**a**) typical twisted SiO_x_ with front ellipse-shaped large head, (**b**) new SiO_x_ formation from two different SiO_x_ parts, and (**c**) fixed balloon whisk-like SiO_x_ formation originating from surplus thermal and partial pressure.


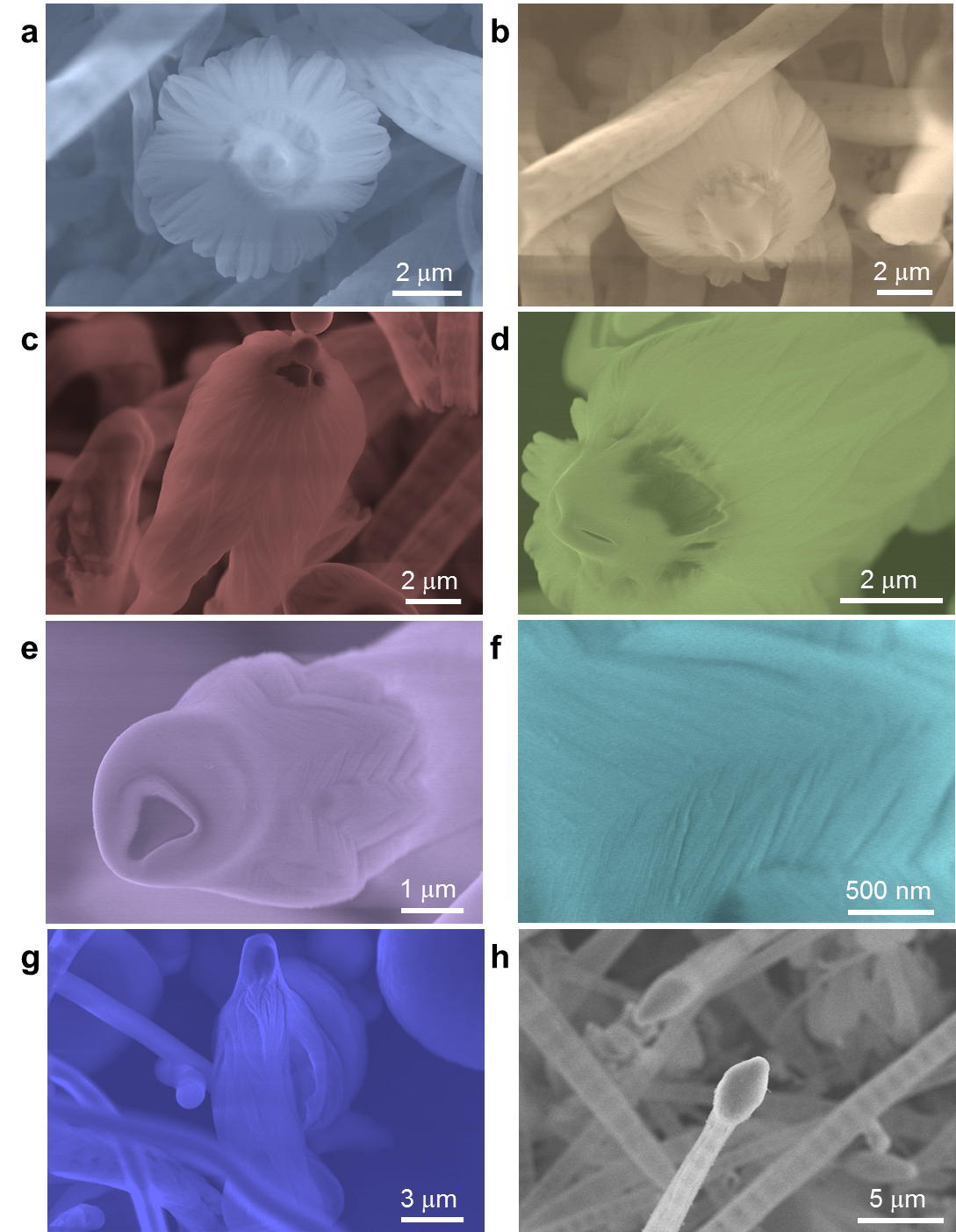


**Figure S5.** **Sequence of SEM images revealing the formation of 1D tubular SiO_x_ from flower-like morphology.**


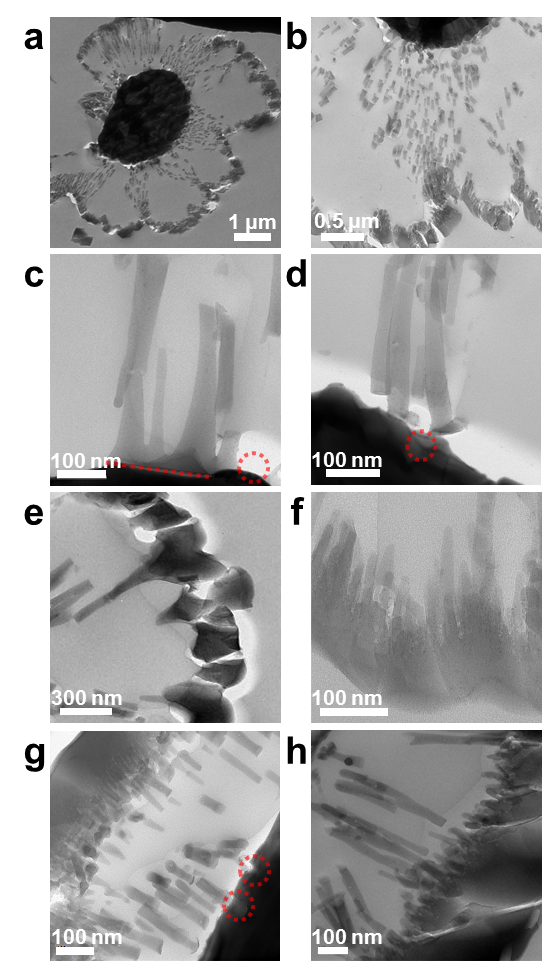


**Figure S6. TEM images of inside flower-like SiO_x_**: (**a**) typical flower-like image of SiO_x_, (**b**) enlarged TEM image of (a), (**c,d**) growth of nano-scaled SiO_x_ at the Sn lump, (**e,f**) growth of nano-scaled SiO_x_ at the SiO_x_ walls, (**g**) TEM image indicating both the Sn lump and SiO_x_ walls, and (**h**) typical newly produced SiO_x_ around SiO_x_ walls. The red circles indicate where SiO_x_ nanowires exist on the Sn surface.


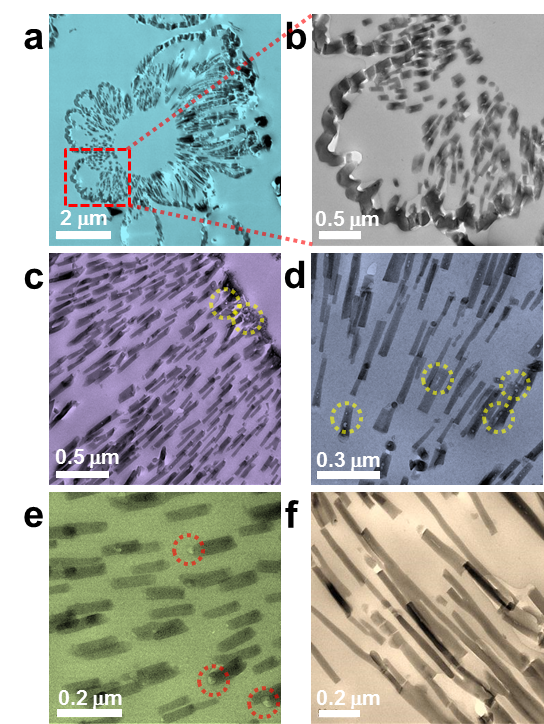


**Figure S7. Arrangement inside flower-like SiO_x_**: (**a**) typical flower-like SiO_x_ image, (**b**) enlarged TEM image of inside aggregated SiO_x_ loops, and (**c–f**) newly produced SiO_x_ nanowires. The yellow and red circles indicate where nanoholes and nanotubes, respectively, exist in the SiO_x_ nanowires.


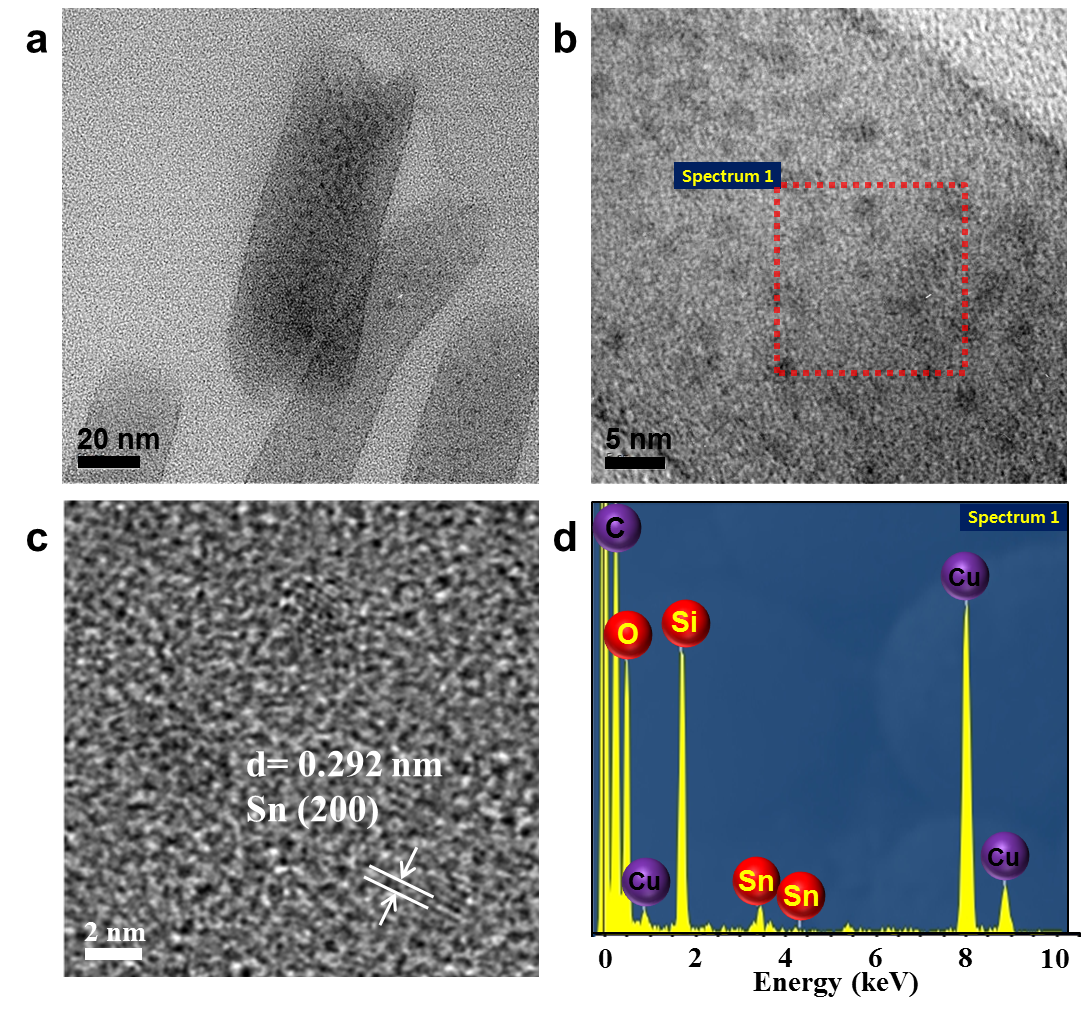


**Figure S8. Microstructure and elemental composition of tubular SiO_x_ structures**: (**a**) typical Sn-embedded SiO_x_, (**b**) enlarged Sn-embedded SiO_x_, (**c**) HRTEM image of (b), and (**d**) EDX spectra corresponding to the sample.


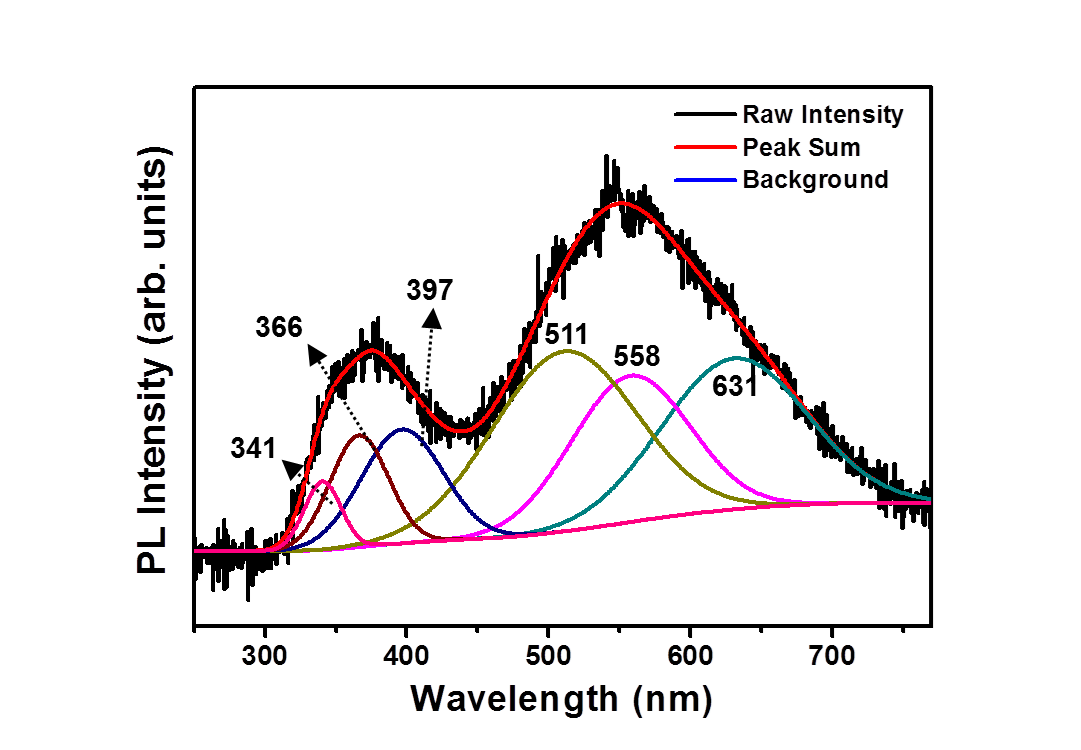


**Figure S9.** **PL spectra of Sn-embedded SiO_x_ with Gaussian fit.**

**Movie S1.** Real-time Sn embedment process into SiO_x_ tubes (double click to play).

**Movie S2.** Emission of SiO_x_ tube (double click to play).

**Movie S3**. Emission of tubular SiO_x_ flower (double click to play).
